# Environmental Impact of Geosynthetics in Coastal Protection

**DOI:** 10.3390/ma14030634

**Published:** 2021-01-29

**Authors:** Philipp Scholz, Ieva Putna-Nimane, Ieva Barda, Ineta Liepina-Leimane, Evita Strode, Alexandr Kileso, Elena Esiukova, Boris Chubarenko, Ingrida Purina, Franz-Georg Simon

**Affiliations:** 1 BAM Bundesanstalt für Materialforschung und-prüfung, 12200 Berlin, Germany; philipp.scholz@bam.de; 2 Latvian Institute of Aquatic Ecology, 1007 Riga, Latvia; ieva.putna@lhei.lv (I.P.-N.); ieva.barda@lhei.lv (I.B.); ineta.liepina@lhei.lv (I.L.-L.); evita.strode@lhei.lv (E.S.); ingrida.purina@lhei.lv (I.P.); 3 Shirshov Institute of Oceanology, Russian Academy of Sciences, 117997 Moscow, Russia; aleksandr.kileso@gmail.com (A.K.); elena_esiukova@mail.ru (E.E.); chuboris@mail.ru (B.C.); 4 Immanuel Kant Baltic Federal University, 236041 Kaliningrad, Russia

**Keywords:** geosynthetics, geotextiles, dynamic surface leaching test, artificial ageing, marine littering

## Abstract

Geosynthetic materials are applied in measures for coastal protection. Weathering or any damage of constructions, as shown by a field study in Kaliningrad Oblast (Russia), could lead to the littering of the beach or the sea (marine littering) and the discharge of possibly harmful additives into the marine environment. The ageing behavior of a widely used geotextile made of polypropylene was studied by artificial accelerated ageing in water-filled autoclaves at temperatures of 30 to 80 °C and pressures of 10 to 50 bar. Tensile strength tests were used to evaluate the progress of ageing, concluding that temperature rather than pressure was the main factor influencing the ageing of geotextiles. Using a modified Arrhenius equation, it was possible to calculate the half-life for the loss of 50% of the strain, which corresponds to approximately 330 years. Dynamic surface leaching and ecotoxicological tests were performed to determine the possible release of contaminants. No harmful effects on the test organisms were observed.

## 1. Introduction

Geosynthetics are widely used in coastal protection. Their application areas are soil reinforcement, the stabilization of ballast layers, filtration, the waterproofing of dams and canals, and scour protection (e.g., for piles of offshore wind energy plants). The application of geosynthetics in coastal protection has huge economic benefits, such as savings via substitutions of or reductions in selected soil materials, ease of installation, increased speed of construction, life cycle cost savings through improved performance (by increased longevity or reduction in maintenance), and improved sustainability in terms of conserving natural environments as compared to alternative designs [1,2]. It is commonly accepted that geosynthetics which are adequately stabilized with antioxidants (e.g., sterically hindered amines) will last in underwater constructions with limited oxygen supply and temperatures at constantly low levels for at least 100 years.

However, after the end of service lifetime, geosynthetics could be a source of plastic debris in aquatic systems if the construction which the geosynthetic is a part of is not dismantled. Further, additives which are needed as plasticizers or antioxidants could be emitted, with detrimental influence on the environment [3]. The loss of additives is intimately related to the aging of the geosynthetic products. These are the reasons that public authorities are concerned about the approvability of engineering projects using geosynthetics in aquatic systems.

The long-term stability of geotextiles is usually investigated with relation to mechanical stability, which must fulfill certain requirements after aging. Various methodologies are available (e.g., elevated temperatures or increase in oxygen pressure) to accelerate aging in the laboratory [4]. Mechanical properties, such as tensile strength, investigation of chemical oxidation reactions by infrared spectroscopy, and the residual content of stabilizers are typical parameters tested on aged samples [5]. The investigation of the possible environmental impact of the application of geosynthetics in aquatic systems is therefore hardly possible with virgin polymer material. Consequently, polymers must be artificially aged, which is best accomplished with environmental simulation chambers enabling accelerated ageing. In the case of geosynthetics in hydraulic engineering besides oxidation, mechanical stress (e.g., by tidal and wave action, abrasion by sand) and microbiological interactions (the formation of biofilms, etc.) [6] play significant roles and must be considered.

There are only a few investigations on the degradation behavior of geotextiles in marine environments [7,8]. According to these, exposure to UV light has a higher impact on the material properties in comparison to seawater immersion and tidal action. The importance of the stabilization of the polymers was strengthened. It can be expected that the degradation processes of geotextiles are similar to the processes of other plastics reaching the marine environment because they are made from the same types of polymers. Plastic waste exposed to environmental conditions begins to degrade slowly under the impact of temperature and UV radiation [9], generating a large number of macro-, micro­, and nano-particles. These particles are freely transported by water flows and have adverse effects on the environment [10,11]. One of the key factors which determines the fate of microplastics in the environment is the density of polymers. The specific density of microplastic can vary significantly depending on the polymer type, technological processes of its production, additives, weathering, and biofouling [12,13]. With time, most floating plastics become negatively buoyant due to both biofouling and the adherence of denser particles and sink to the sea floor [13,14]. Thus, the seabed becomes the ultimate repository for microplastic particles and fibers [15,16]. The evaluation of the contamination level is complicated, not only because of the difficulty of the sampling of sea bottom sediment, but also due to the difficulty of the extraction of small plastic particles from marine deposits.

The project Environmental Impact of Geosynthetics in Aquatic Systems (EI-GEO) [17] aims at the investigation of whether geosynthetics in hydraulic engineering applications could be a source of microplastic or other contaminants in the aquatic environment. Whereas the behavior of geosynthetics in landfill engineering has been well studied and documented for decades [18], little is known regarding applications such as coastal protection or scour protection for off-shore wind energy plants. However, due to the rapid expansion of offshore wind energy, rising water levels, and more extreme weather conditions as a result of climate change, more and more hydraulic engineering projects will be realized in the future.

Construction with geosynthetics boasts various advantages, but it has to be ensured that there is no negative environmental impact from the application of geosynthetics in hydraulic engineering. It is expected that any effect will be visible only in the long term because the virgin raw material used for the production of geosynthetics has almost no release of particles or substances relevant to the environment [19].

Partly from improper material selection and partly from non-professional handling, debris from geosynthetic material can be found on the shore today. Therefore, a field study with sampling and monitoring was performed and the magnitude of this pollution was evaluated (objective 1). Further, an accelerated ageing method was performed to derive the requirements for geosynthetics in hydraulic engineering. The testing of mechanical properties was performed with virgin and artificially aged geosynthetics (objective 2). Finally, leachates of artificially aged geosynthetics were used in ecotoxicological tests, which are essential tools to evaluate the environmental impacts of the pollutants released by geosynthetics during ageing (objective 3).

## 2. Materials and Methods

The applications of geosynthetics in hydraulic and coastal engineering such as revetments, dyke constructions, or geotextile containers for scour prevention are described in detail elsewhere [1]. For the present study, a multifunctional geotextile for separation, filtration, and protection made of white polypropylene was selected as a test material for the investigations. The mass per unit area was 600 g m^−2^, the thickness was 5 mm, and the water permeability was 3 × 10^−2^ m s^−1^. The material, produced in Germany, is commercially available and widely used for geomembrane protection or for the production of sand container bags.

### 2.1. Accelerated Ageing Using Autoclave Test

Autoclave tests following DIN EN ISO 13438:2005 (method C) [19] were performed under a pure oxygen atmosphere with pressures between 10 and 50 bar, at temperatures between 30 and 80 °C, and with durations in the range of 14 to 143 days. An overview on the performed ageing experiment is given in Table 1. It is important to notice that the test specimens were completely immersed in tap water and the exposure of autoclaves was carried out based on the time-dependent degradation of the mechanical properties of the polypropylene geotextiles. Five PP specimens (250 × 50 mm^2^) were placed in the autoclaves in tap water. The use of artificial seawater was not possible due to the risk of chlorine-induced corrosion at high oxygen pressures. In order to reach thermal equilibrium, the autoclaves were left for 48 h in electronically controlled heating systems before the start of the tests. Hence, single specimens were removed in succession after different ageing periods. Then, the tensile strength was determined accordingly. Two measurements were carried out for each duration of aging. All the tensile test measurements were performed with a Zwick tensile testing machine (Zwick-Roell, Ulm, Germany) (ZPM Model 1464 with testXpert II software (Version 3.31, Zwick, Ulm, Germany)) with a 5 kN force sensor. The tensile tests were performed in an air-conditioned environment at 23 °C and a relative humidity of 50%. For the tensile test measurements, a clamping length of 50 mm and a test speed of 50 mm/min were chosen. Each sample was attached to a sandpaper to avoid sliding during the tensile test.

**Figure 1:**
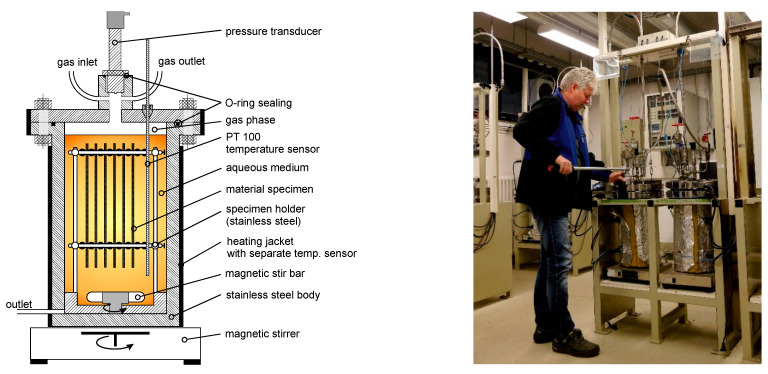
Schematic view of autoclave test equipment (**left**), closing the cover plate of the autoclaves (test rig with two autoclaves).

Figure 1 shows a sketch of the autoclave test equipment along with all the instruments and monitoring devices used. The temperature and the pressure were observed and recorded every 15 min using an electronic data recorder (Eurotherm 6100) (Eurotherm, Limburg, Germany). The temperature of the autoclave was controlled by an external heating jacket with a separate PT100 temperature sensor connected to a PID temperature controller (Eurotherm 2216E) (Eurotherm). The heating power line was equipped with an electrical contact controlled by the internal temperature monitoring to prevent overheating of the system. The safe and reliable operation of the autoclaves requires the control and monitoring of the relevant parameters, especially for long-term experiments. All the relevant instruments and transducers were calibrated in order to obtain reliable and reproducible results.

### 2.2. Dynamic Surface Leaching Test

Dynamic surface leaching tests (DSLT) were performed on the geosynthetic materials according to the CEN/TS 16637-2 leaching method [20]. The DSLT corresponds to a tank test for the assessment of the surface-dependent release of dangerous substances and is suitable for monolithic construction products. The test specimens were eluted using demineralized water at a defined water/surface ratio (L/A) and a water exchange at several fixed time intervals (6 h, 1 d, 36 d). The L/A ratio was set to 80 L/m^2^ in CEN TS 16637-2, but can be reduced to 25 L/m^2^ for plate-like products. Tests were performed at 23 ± 2 °C, room humidity 50 ± 5%, in the darkness. Two plates were eluted per coating system to obtain enough eluate volume for all the ecotoxicological tests. Each plate was individually placed in a tank and the eluates of the same fraction were combined and well mixed before aliquoting them for ecotoxicological analysis.

### 2.3. Ecotoxicological Testing

Internationally agreed and accepted ecotoxicity test methods have been performed to demonstrate the impact of chemicals and other pollutants on the environment and determine the potential damage to organisms and the function of ecosystems [21,22,23]. Ecotoxicity tests consisted of two acute and one chronic test with organisms from different levels of aquatic food chains. The ecotoxicity test conditions, growth media, dilutions, and replication are summarized in Table 2. The test eligibility criteria for the *Daphnia magna* test is ≤10% immobile organisms in the control treatment and an ≥80% survival for the *Hyalella Azteca* test. For the *Desmodesmus subspicatus* test, control batch absorption measurements should indicate the exponential growth of algal cells, the variation coefficient (CV) of the growth rate in the control replicates should not exceed 5%, and the pH in the control should not increase during the test by more than 1.5 relative to the pH of the growth medium.

### 2.4. Continuous Visual Scanning (Field Study)

Since the fragments of plastics and geosynthetic materials were unevenly distributed on the beach, the use of a selective area technique for their search—such as, for example, for anthropogenic debris [24] and microplastics [25]—will not yield results. To analyze the pollution of the beaches at the Southeastern Baltic within the Kaliningrad Oblast (Russia), a continuous visual scanning technique [26] was applied which assumes a continuous passage of a group of several people along the entire coastline, covering the entire width of the beach from the shoreline to the foredune (or cliff).

The width of the beaches of the Kaliningrad Oblast ranges from almost 0 to 188 m and the average value is 30 m, so the group usually included three people. The beach was divided into three control strips, each member of the group controls his strip and even tries to capture the edge of the neighboring zone for a complete scan of the entire beach. During the day, the group could walk 7–10 km, and such monitoring surveys were carried out in 2018.

Each detected plastic or geosynthetic fragment with a size larger than 3–5 cm was attributed to the different type of origin (see Results section), dimension scale (length and area), number of the coastal subsection where this sample was found, and position on the beach (in % of the beach width). Next, photographs were taken and, if necessary, the sample was saved for further laboratory analysis.

## 3. Results

### 3.1. Field Study on Kaliningrad Oblast Shore (Russia)

**Figure 2:**
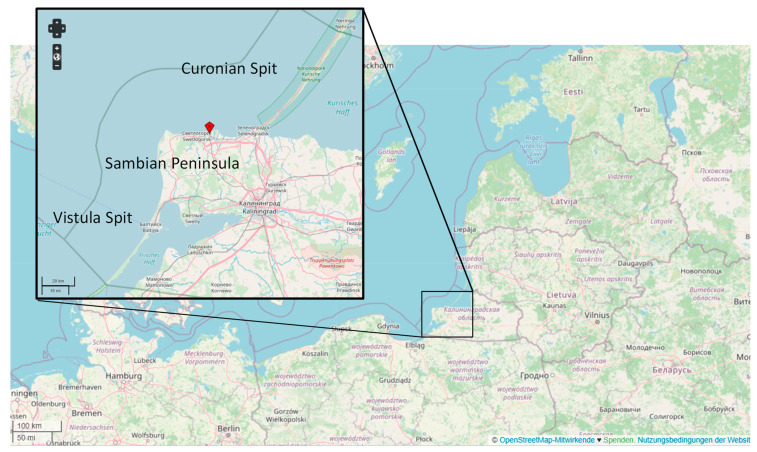
Shoreline of the Kaliningrad Oblast (Russia) in the Baltic Sea including the Sambian Peninsula (quadrangle). Source: OpenStreetMap.

During the surveys of the beaches of the Kaliningrad Oblast (Figure 2) in 2018, a large amount of remnants of geosynthetic materials that are used in coastal protection structures [27] were found. In addition, there was extensive contamination from other building support materials—e.g., geotextile FIBC (Flexible Intermediate Bulk Container) bags (“big bags”) and the remains of fishing nets, ropes, and car tires.

In 2018, 3485 samples were collected from the beaches which, by origin, belonged to several types of materials: geotextiles, geocells, geogrids, plastic coating from gabions, and geotextile big bags. The integral amount of remnants of geotextile objects was more than 190 m^2^ and the integral length of the geotextile braids from gabions coating was about 100 m [28].

The occurrence of geosynthetic remnants varies greatly along the entire shore of the Kaliningrad Oblast. The northern shore of the Sambia Peninsula accounts for 66% of the remains found, 31% for the beaches of the Curonian Spit National Park, and only 3% was found on the beaches of the western shore of the Sambia Peninsula and the Vistula Spit. Among the remains of geosynthetic materials found, the largest number was braid from gabions (44%) and geocontainers (43%), pieces of geotextile accounted for only 12%, and the remaining 1% was made up of remnants of geocells and geogrids.

The performed primary statistical analysis on the occurrence of the number of pieces per 1 kilometer for various morphodynamic segments of the coast of the Kaliningrad Oblast (Vistula Spit, western and northern shores of the Sambia Peninsula, Curonian Spit) showed that the main pollution occurs on the northern shore (see Table 3). Considering the average size of one piece of geotextile (0.9 m^2^), gabion coating (7.4 cm), big bag (0.3 m^2^), and geocell (0.06 m^2^), it is obvious that the remnants of geotextile and “big bags” were the mostly visible litter on the beach.

This fact that the northern shore of the Sambian Peninsula is mostly littered correlates well with the location of engineering structures using geosynthetic materials, most of which are located on the northern shore of the Sambian Peninsula [27]. In addition, the main accumulation of residues of geosynthetic materials is observed in the areas adjacent to these engineering structures. On the Curonian Spit (north from the Sambian Peninsula), a large amount of geosynthetic remnants was also found, which were probably brought here by alongshore currents [29]. The occurrence of residues on the Vistula Spit (south from the Sambian Peninsula) and on the western coast of the Sambia Peninsula is low due to the current structure in the eastern part of the Gulf of Gdansk [30].

Gabion coating was found quite often (see Table 3). This came from the plastic coating of the wire used for the gabion’s support structure. Obviously, this coating is not weatherproof. A support structure made of stainless steel or Zn-plated wires would not need a plastic coating but is, however, more expensive. Geotextile remnants came from partly destroyed coastal protection structures which stay without proper maintenance during long time. Geocells were found rarely, they were from several locations, where storm events destroyed lawn on the slopes of foredune wall prepared using geocells. Debris from big bags was found often as well. However, these woven geotextiles are rather used for transport of building materials or short-term applications than for coastal protection systems. Occurrence can therefore be attributed to improper waste management.

### 3.2. Tensile Tests after Accelerated Ageing Using Autoclave Test

The elongation and force of break of the test specimens were measured on a tensile testing machine. The retained elongation R_ε_ at break is measured as a function of time (and temperature and oxygen pressure) and is expected to be influenced by the ductile–brittle change which is a service lifetime criterion for the geotextile. The retained elongation R_ε_ is defined as follows:

R_ε_ = 100% ε_e_/ε_c_, (1)

with ε_e_ then initial elongation at break and ε_c_ the elongation of the exposed specimen.

**Figure 4:**
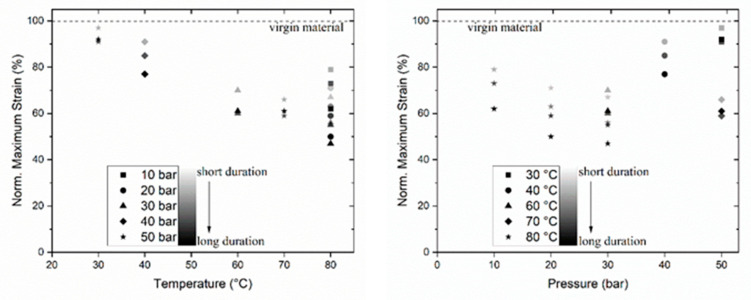
Retained elongation R_ε_ measured after exposure in autoclaves as a function of temperature (**left**) and pressure (**right**). The duration of exposure is visualized by the gray scale of the symbols. Note that at 80 °C experiments at three different pressures (10, 20, and 30 bar, different symbols) were performed.

The results are displayed in Figure 4. It is clearly visible that increasing temperature leads to a more pronounced decay of the mechanical properties. The loss of retained elongation proceeds with the duration of the exposure, which is visualized in Figure 4 by different gray scales of the respective symbols (bright to dark). The influence of pressure is lower. Experiments performed at 40 and 50 bar show higher values for retained elongation because the temperature was 30 °C and 40 °C, respectively.

The aging of polymers is caused by oxidation. The thermo-oxidation of PP can be defined as an in-chain radical mechanism. The latter generates hydroperoxides more rapidly than they decompose, which strengthen its strong auto-accelerating character. A detailed description of the oxidative aging of polymers is given by Verdu [31]. The accelerated ageing in the autoclaves is a function of temperature and pressure with a (pseudo-)first-order rate constant k (s^−1^). The temperature and pressure dependence of the oxidation reaction can be approximated by an modified Arrhenius equation (consideration of pressure dependence) [32,33]:

(2) $$\ln\frac{\varepsilon_{e}}{\varepsilon_{c}}\sim \ln\frac{c_{0}}{c}=A\;\exp\left(\frac{-E_{a}+C\;p}{R\;T}\right)=k\;\left(T,\;p\right)\;t,$$

with frequency factor A (s^−1^), activation energy E_a_ (J mol^−1^), pressure factor C (J mol^−1^ bar^−1^), universal gas constant R, and temperature T (K).

The term ln c_0_/c is usually related to the fate of a substance in a chemical reaction. Here, it is approximated by the loss of mechanical properties and describes the progress of the oxidation and thus degradation of the material without knowing exact concentration of oxidized and non-oxidized polymer material. The experimental data displayed in Figure 4 were fitted with the Solver module in Microsoft Excel (solver method GRG non-linear) (Office 365 for Enterprise). Starting values for activation energy E_a_ (80,000 J mol^−1^) and frequency factor A (6 × 10^8^ s^−1^) were taken from the literature [34]. As a result, k (T, p) was fitted to 0.5 s^−1^ at T = 298 K and p_O2_ = 0.21 bar. The half-life τ at 298 K and 0.21 bar oxygen pressure, i.e., the time were 50% of the mechanical properties are lost under ambient conditions, can be calculated from ln2/k.

τ = ln2/k = 330 years(3)

This result is in the same order of magnitude as the results from Hausmann et al. for woven polypropylene geotextiles [34] (483–795 years). Fitted pressure factor C was 146 J mol^−1^ bar^−1^, so the activation energy E_a_ in the exponential tern in Equation (2) is reduced by 7300 J mol^−1^ (<10%) at 50 bar oxygen pressure in the autoclave experiment. As stated above, temperature has the strongest influence on the accelerated ageing in the autoclaves, even at highest possible pressure of 50 bar. However, it must be mentioned at this point that the samples are immersed in tap water so that the samples are exposed to the dissolved oxygen in water which is proportional to the partial pressure of oxygen above the liquid (Henry’s law). Henry’s law solubility constant is substance specific and a function of temperature. An equation to calculate the concentration of dissolved oxygen c_aq_ in water between 273 and 616 K and pressures up to 60 bar was presented by Tromans [35] and reviewed by Sander [36]. For 50 bar and 353 K, the c_aq_ is 3.97 × 10^−2^ mol kg^−1^.

### 3.3. Ecotoxicity Tests

To evaluate the geosynthetic leachate ecotoxicity, a combination of bioassays was applied—both acute and chronic tests and organisms representing two trophic levels were used. Such an approach has advantages over individual component analysis and testing because it can disclose mixture effects.

**Figure 5:**
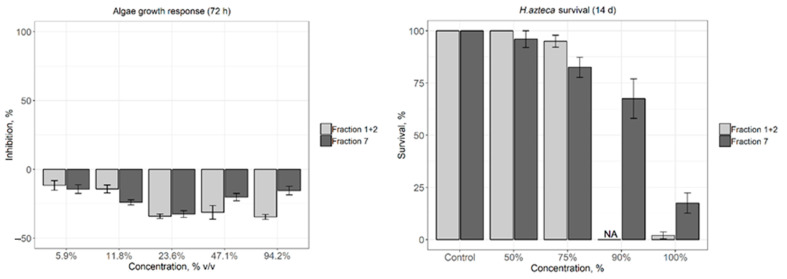
Algae growth response after 72 h (optical density measurements at 680 nm, **left**), *Hyalella. azteca* survival after 14 days (**right**). (NA: not analyzed, right).

The algae growth inhibition test was conducted at five volume/volume percent concentrations—5.9%, 11.8%, 23.6%, 47.2%, and 94.3%. Inhibition is evaluated by the reduction in specific growth rate relative to the cultures of the control. Samples Fraction 1 + 2 and Fraction 7 after 72 h exposures did not indicate algae growth inhibition even at the highest test concentration (Figure 5).

**Figure 6:**
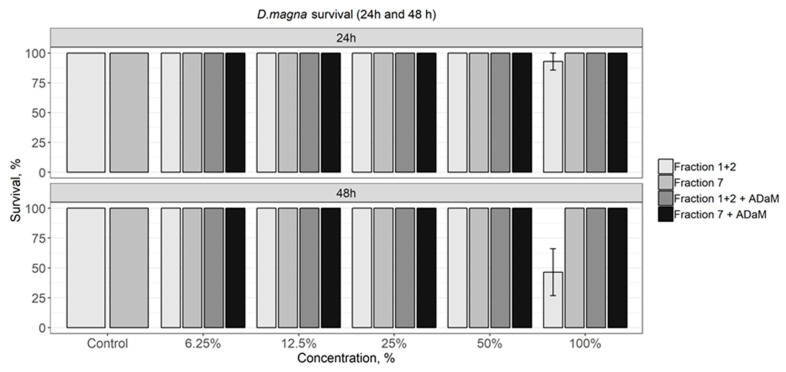
Survival of *Daphnia magna* after 24 h and 48 h.

The results of an acute *Daphnia magna* test showed the toxicity of Fraction 1 + 2 only at 100% concentration, causing 7.1% daphnia mortality after 24 h and 54% of cladocera mortality after 48 h exposure (Figure 6). However, there was no toxic effect observed when ADaM media microelements were added to the highest concentration. Fraction 7 did not cause any effects on *D. magna* survival during the test.

Although the acute ecotoxicity test results of amphipod *Hyalella azteca* showed the higher toxicity of Fraction 1 + 2 than Fraction 7, no significant differences in toxicity between both samples after 14 days exposure were detected (Figure 5). In the 100% concentrate samples, an 88% mortality of amphipods was detected in Fraction 1 + 2 after 48 h exposure, while toxicity of Sample 7 increased only after one-week exposure. LC_50_ for Fraction 1 + 2 was 83%, while Fraction 7—LC_50_ was at 89%.

Measurements of pH showed an increase by 0.5 units after the 14-day test period, while the oxygen concentration stayed uniform more than 8.00 mg/L all test period. Ammonium concentration during the test did not reach higher than 20 mg/L (ISO 16303:2013 standard mentioned 96 h LC50 ammonium could be 20 mg/L to >200 mg/L [21]).

## 4. Discussion

The loss of additives, such as plasticizers and antioxidants, during the ageing of geotextiles potentially can add to the concentrations of hazardous substances in the water. This is discussed in a study from South Korea, where more than 200 different chemicals were identified in plastic marine debris and respective new products [37]. Another consideration is that base structure forming polymers gradually degrades to microplastic particles, and as such can be ingested by heterotrophs or interfere with algal photosynthesis [3]. However, ecotoxicological test results in this research did not show significant toxicity of geotextile leachates to water organisms. In case of microalgae, the test samples showed even nutritive properties, as an increase in microalgae concentration was observed during the 72 h of the test. Currently, there is limited research in the field of geosynthetic ecotoxicity, but a study evaluating the environmental safety of construction products also found that geosynthetic PET multifilament yarns and polyamide monofilament with PP fleece coating, have low toxicity [38]. Results indicate that the algae species *Desmodesmus subspicatus* that were also used in our study are slightly less sensitive than the algae *Raphidocelis subcapitata* and daphnia [39].

A concentrated sample of Fraction 1 + 2 (100%) caused mortality of *Daphnia magna.* However, if test sample was spiked with minerals from ADaM growth media, no mortality was observed. No mortality was observed in other test sample dilutions, neither in Fraction 1 + 2, nor Fraction 7. The results indicate that deionized water used in DSL tests might bias the ecotoxicity tests by adding hypoosmotic stress to low toxicity of test media. Concentrated samples (100%) of Fraction 7 did not caused mortality of organisms. These results suggest that the toxicity of additives is decreasing with time and dilution, also indicating that osmotic stress alone does not cause mortality [40].

A lethal concentration (LC_50_) was calculated only for amphipods *Hyallela Azteca*. However, the LC_50_ at 83% and 89% concentrations can be considered as very low toxicity [41]. As geotextiles in hydraulic engineering are exposed to intensive water exchange, no toxic effects in the environment will be observed. However, even though within the tests with *Daphnia magna*, *Hyalella aztecal* and *Desmodesmus subspicatus* negative effects were not detected, the risk that long-term harsh climate conditions pose an impact on the release and migration of particles as well as hazardous substances cannot be excluded completely (referring to objective 3).

Service lifetime of geotextiles with state-of-the-art stabilization is far above 100 years, which was shown in the present study with accelerated ageing at elevated temperatures and oxygen pressures. The improper installation of the geotextiles and the lack of service and maintenance after extreme weather events could cause the failure of the engineered structures and, as a result, the pollution of the environment by remnants of geosynthetic materials [42]. The successful application of geotextiles in coastal protection depends on the selection of a suitable material and proper installation and maintenance (referring objective 2).

The field study performed at the shore of Kaliningrad Oblast (Russia) demonstrated that debris from plastic and geotextile materials is found in the environment [27,42]. The remnants of the geosynthetic materials are found not only at the beaches of the Kaliningrad Oblast, but at the neighboring beaches of Lithuania [43]. Some of the found objects could be attributed to unsuitable material selection (gabion coating) or improper waste management. Considering that any damage, even partial, of the coastal protective constructions using geosynthetic material could lead to the littering of the beach or the sea, specific attention is needed for the maintenance of such constructions (referring objective 1).

## Figures and Tables

**Figure 3:**
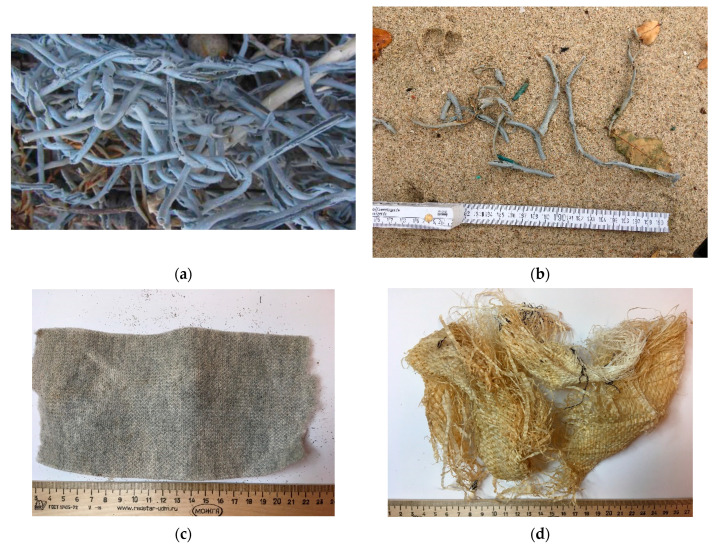
Photographs of samples collected during the field study: (**a**) + (**b**): aged plastic coating of wires in gabions’ (**c**) debris from geocell; (**d**): debris from big bag.

**Table 1:** Duration of accelerated ageing in autoclaves in days at 5 different temperatures and pressures.

p (bar)	Temperature (K)				
	303	313	333	343	353
10	-	-	-	-	14, 44, 61
20	-	-	-	-	27, 54, 82, 140
30	-	-	70, 102, 144	-	28, 38, 48, 77
40	-	70, 101, 143	-	-	-
50	70, 101, 143	-	-	70, 101, 143	-

**Table 2:** Ecotoxicity test conditions summary.

Standard	ISO 6341:2012 [21]	ISO 16303:2013 [22]	ISO 8692:2012 [23]
Test organisms	*Daphnia magna*	*Hyalella azteca*	*Desmodesmus subspicatus*
Test duration	48 h	14 days	72 h
Temperature	20 ± 1 °C	23 ± 1 °C	23 ± 2 °C
Growth media	ADaM *	ADaM	BG-11
Test chamber size	6 vial plates	400 mL low form beakers	300 µL
Test volume	15 mL	250 mL	265 µL
Age of test organisms	Less than 24 h old	11 days old at test initiation (1 to 2 day range in age)	Algae culture in exponencial growth phase
Organisms per test chamber	7	10	5 µL (10^4^ cells)
Replicates per treatment	4	4	6
Test concentrations	(100%; 50%; 25%; 12.5%; 6.3%	100%; 75%; 50%; 25%; 12.5%; 6.3%; 3.1%	5.9%, 11.8%, 23.6%, 47.2%, 94.3%
Feeding regime	No	YCT food, fed 0.5 mL daily/chamber	Concentrated BG11 (10 µL)/vial in beginning of test
Endpoints	Mortality	Survival (optional, growth by dry weight or length)	Growth inhibition
Reference toxicant	K_2_Cr_2_O_7_ 24 h LC 50 0.81 mg/L	CdCl_2_ (Cd 96 h LC50 = 0.007 mg/L), CuSO_4_ (Cu 96 h LC 50 = 0.24–0.33 mg/L)	ISO mentioned intercalibration K_2_Cr_2_O_7_ 72 h EC 50 = 0.84 mg/L

* ADaM: Aachener Daphnia Medium.

**Table 3:** Occurrence of residues of geosynthetic materials and other large debris in pieces per 1 running kilometer of the coastline in various morphodynamic segments of the Baltic shore of the Kaliningrad Oblast by surveys in 2018.

Morphodynamic Segments of the Shore	Geotextile	Gabion Coating	“Big Bags”	Geocell
Vistula Spit	0.01	0.13	0.25	0
Western shore of the Sambian Peninsula	0	0.18	0.15	0
Northern shore of the Sambian Peninsula	2.90	9.38	5.98	0.13
Curonian Spit	0.24	1.97	4.26	0.09

Note: Numbers are given in pieces/km, while pieces have very different linear sizes (see Figure 3 for examples).

## Data Availability

Data sharing is not applicable to this article.
